# Supplementary material for: Molecular-channel driven actuator with considerations for multiple configurations and color switching
Source: Nat Commun. 2018 Feb 9;9:590. doi: 10.1038/s41467-018-03032-2 (PMC5807312; doi:10.1038/s41467-018-03032-2)
Supplement: Supplementary file 1 — Supplementary Information [file 41467_2018_3032_MOESM1_ESM.pdf]

Supplementary Information

Molecular-Channel Driven Actuator with Considerations for Multiple Configurations and Color Switching

Jiuke Mu,[1]† Gang Wang,[2]† Hongping Yan,[3] Huayu Li,[2] Xuemin Wang,[4] Enlai Gao,[5] Chengyi Hou,[1] Anh Thi Cam Pham,[4] Lianjun Wu, [4] Qinghong Zhang,[1,6*] Yaogang Li,[6] Zhiping Xu,[5] Yang Guo,[1] Elsa Reichmanis,[2*] Hongzhi Wang[1*] & Meifang Zhu[1]

Affiliations

1 State Key Laboratory for Modification of Chemical Fibres and Polymer Materials, College of Material Science and Engineering, Donghua University, Shanghai, 201620, China

2 School of Chemical and Biomolecular Engineering, School of Chemistry and Biochemistry, School of Materials Science and Engineering, Georgia Institute of Technology, Atlanta, Georgia, 30332, USA

3 Stanford Synchrotron Radiation Light Source, SLAC National Accelerator Laboratory, 2575 Sand Hill Road, Menlo Park, California, 94025, USA

4 Department of Mechanical Engineering, University of Texas at Dallas, Richardson, TX 75080, USA

5 Applied Mechanics Laboratory, Department of Engineering Mechanics and Center for Nano and Micro Mechanics, Tsinghua University, Beijing 100084, China

6 Engineering Research Center of Advanced Glasses Manufacturing Technology, MOE, Donghua University, Shanghai, 201620, China

†These authors contributed equally to this work.

* Correspondence and requests for materials should be addressed to H.Wang. (email: wanghz@dhu.edu.cn), Q.Zhang. (email: zhangqh@dhu.edu.cn) or E. Reichmanis. (email: ereichmanis@chbe.gatech.edu)

Morphology characterization of the PFSA membrane

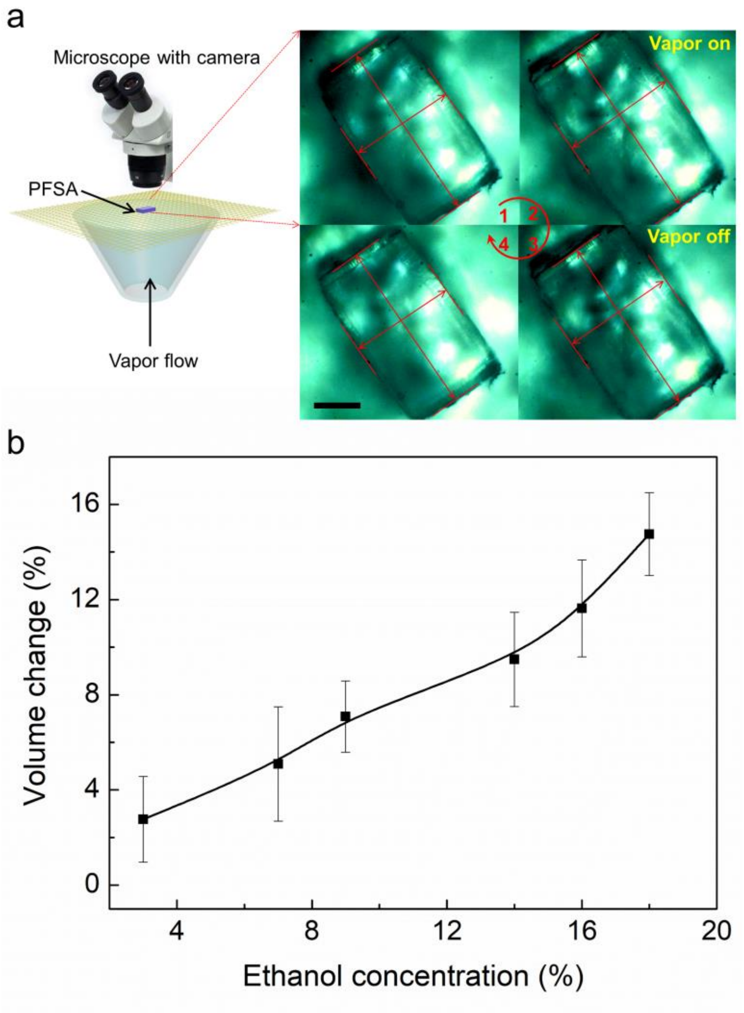

Supplementary Figure 1. The thickness–time dependence plot of the PFSA single-layer membrane under ethanol vapor triggering. a Optical microscope images of a piece of PFSA film (450×750 μm) which shows the changes in length and width of PFSA films in the dry state, and upon exposure to 18% ethanol vapor. (The scale bar is 200 μm) **b** The equilibrium volume change of the PFSA films when exposed to different ethanol vapor conditions.

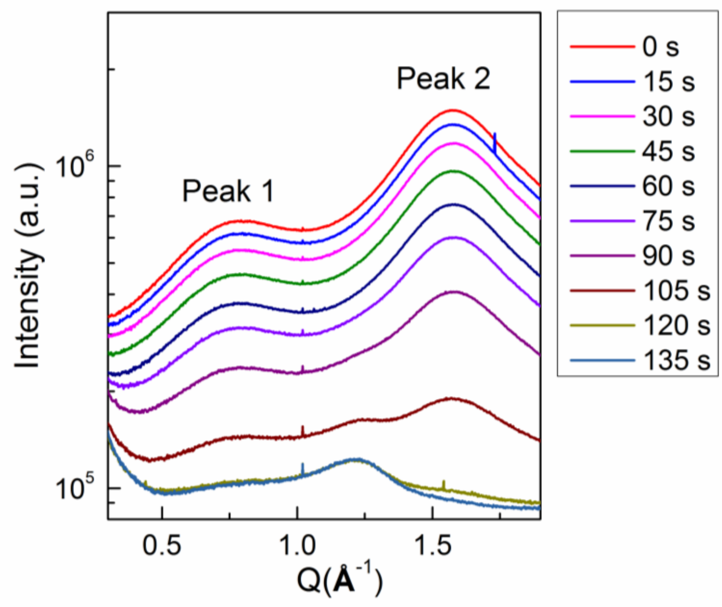

Supplementary Figure 2. The line-cuts of the time-dependent *in-situ* GIWAXS with the ethanol evaporation.

In order to further understand the change in domain ordering during ethanol actuation, we designed a time-dependent 2D GIWAXS, in-situ X-ray scattering experiment so as to monitor the ordering spacing change during de-actuation in real time. The sample was placed on an x-ray scattering stage, which was in a small chamber (~10×10×10 cm^3) fitted with a Kapton window to facilitate X-ray exposure. The x-ray detector used in this experiment has a maximum image acquisition rate of 1 second/frame, however, the required sample exposure time for each frame for sufficient signal to noise needed to be at least 10 seconds. In order to effectively modulate the ethanol evaporation rate to be commensurate with this exposure time, we controlled the opening of the sample chamber and the flow of the helium gas into the chamber (which also helped reduce scattering of the X-rays from residual air). The evaporation and desorption of the ethanol were thereby slowed down, which allowed us to monitor the de-actuation process. This process, however, did not reflect the true, as measured actuator response time.

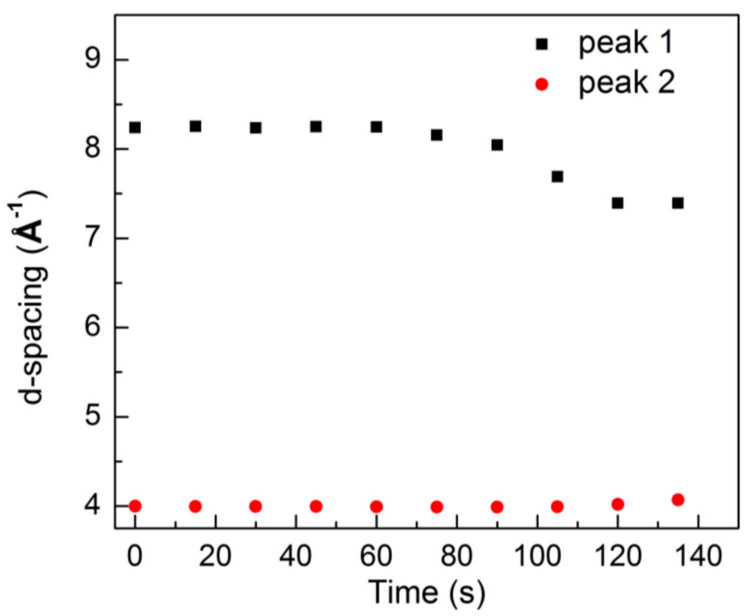

Supplementary Figure 3. The spacing variation of peak 1 and peak 2 calculated from the *in-situ* GIWAXS patterns.

The incorporation of the ethanol molecules into the hydrophilic nano-channels promoted the ordering that results in the scattering peak at ~0.76 Å^{-1} (corresponding to an ordering spacing of ~8.3 Å), and the peak position increased over time with the desorption of the ethanol molecules to ~0.85 Å^{-1} (corresponding to a decreased spacing of 7.4 Å). In theory, this spacing change indicates that the absorption of ethanol could introduce a volume expansion of (8.3/7.4)3-1=40% if other parts of the film are not compressible.

Supplementary Table 1. Summary of different materials and structure for the design of actuators. Note: In each cited paper, we choose the most rapid speed for comparison with our results

Ref.	Response time (s)	Material (Structure)	Type of actuation
1	0.4	PILTf_2N *(Microporous)*	Bending
2	0.38	Cross-linked LC polymers	Bending
3	2	Agarose	Bending
4	3	PVDF/PVA *(Microporous)*	Bending
5	6	PIL-PAA@tissue paper membranes *(Microporous)*	Bending
6	50	PEI/PAA film *(Microporous)*	Bending
7	15	Polyamide-6 substrate and a liquid-crystalline polymer coating	Bending
8	1s	Carbon nanotube	Tensile
9	2.7	Carbon nanotube	Torsional
10	98s	Carbon nanotube	Torsional
11	50	Graphene oxide	Bending

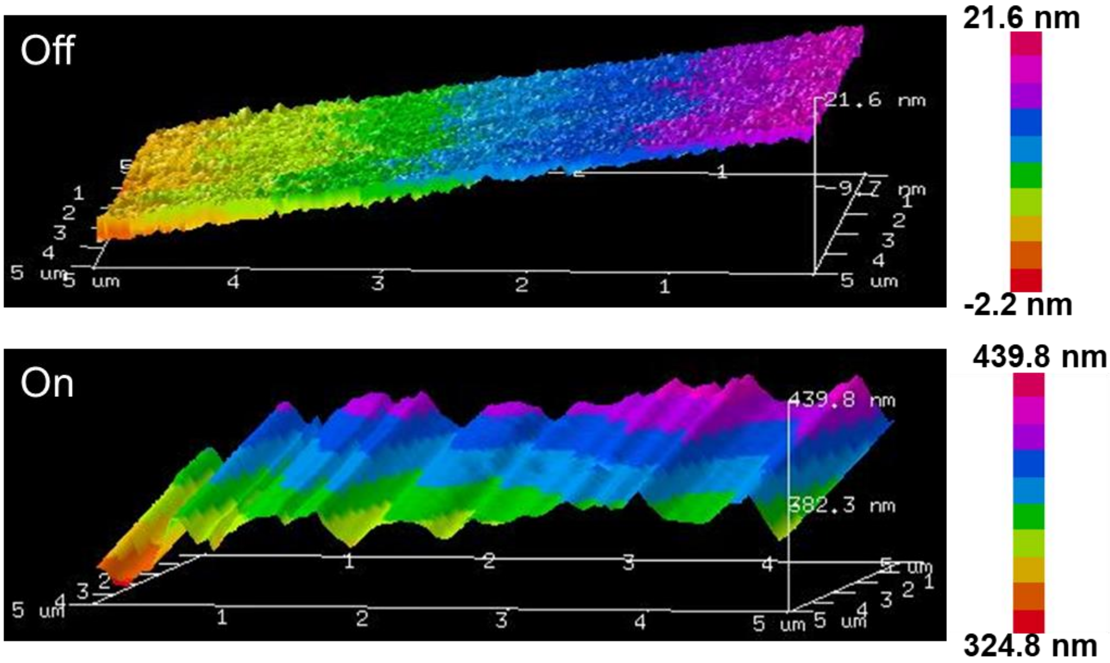

Supplementary Figure 4. ***In-situ*** **AFM height images of the PFSA membrane in the off state (without ethanol vapor evaporation) and on state (with ethanol vapor absorption).** (The scanning size is 5 μm × 5 μm.)

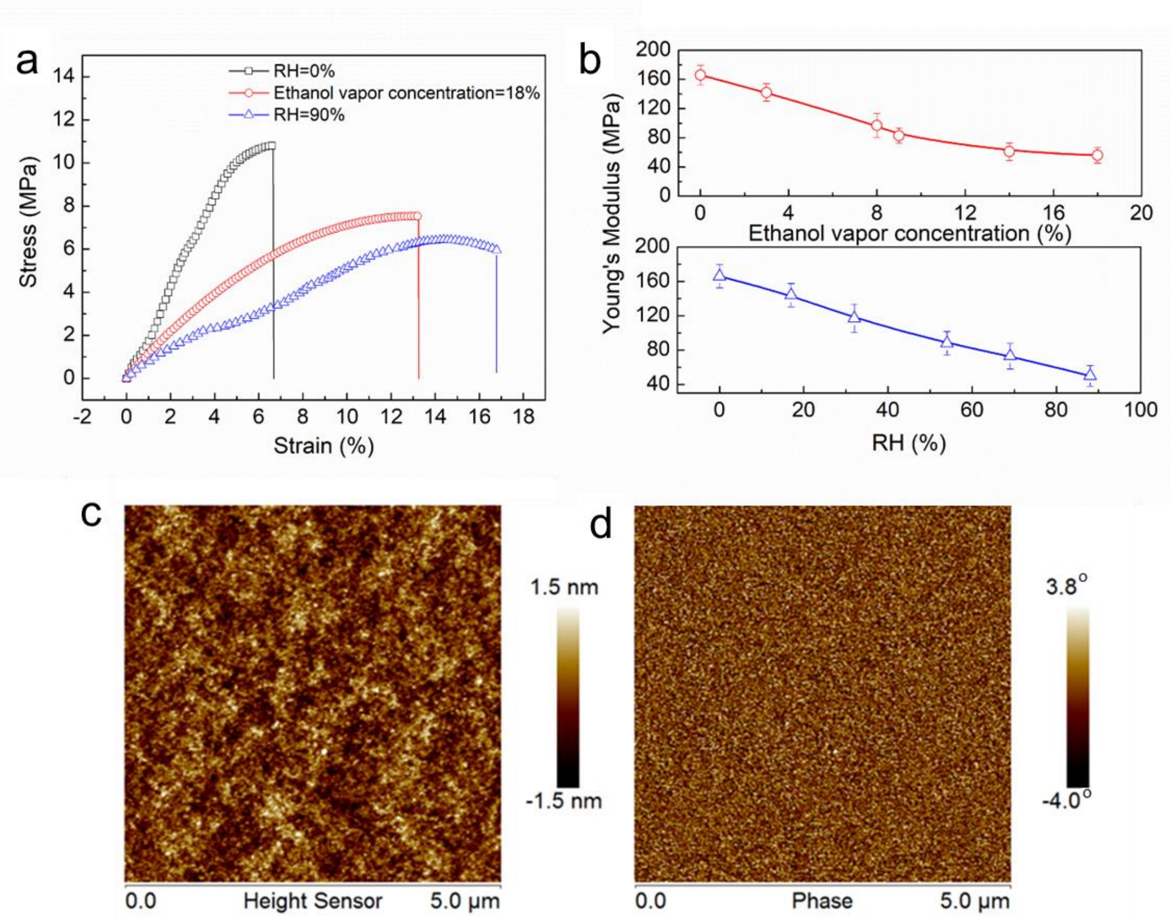

Supplementary Figure 5. Mechanical properties characterization of PFSA membrane. a Engineering stress-strain curves of PFSA films under conditions of no-vapor, 18% ethanol vapor and RH (relative humidity)=90%. **b** Young's modulus as a function of ethanol vapor concentration and RH. **c, d** AFM images of freshly prepared PFSA films exposed to ambient air.

The actuation property of the monolayer PFSA membrane actuator

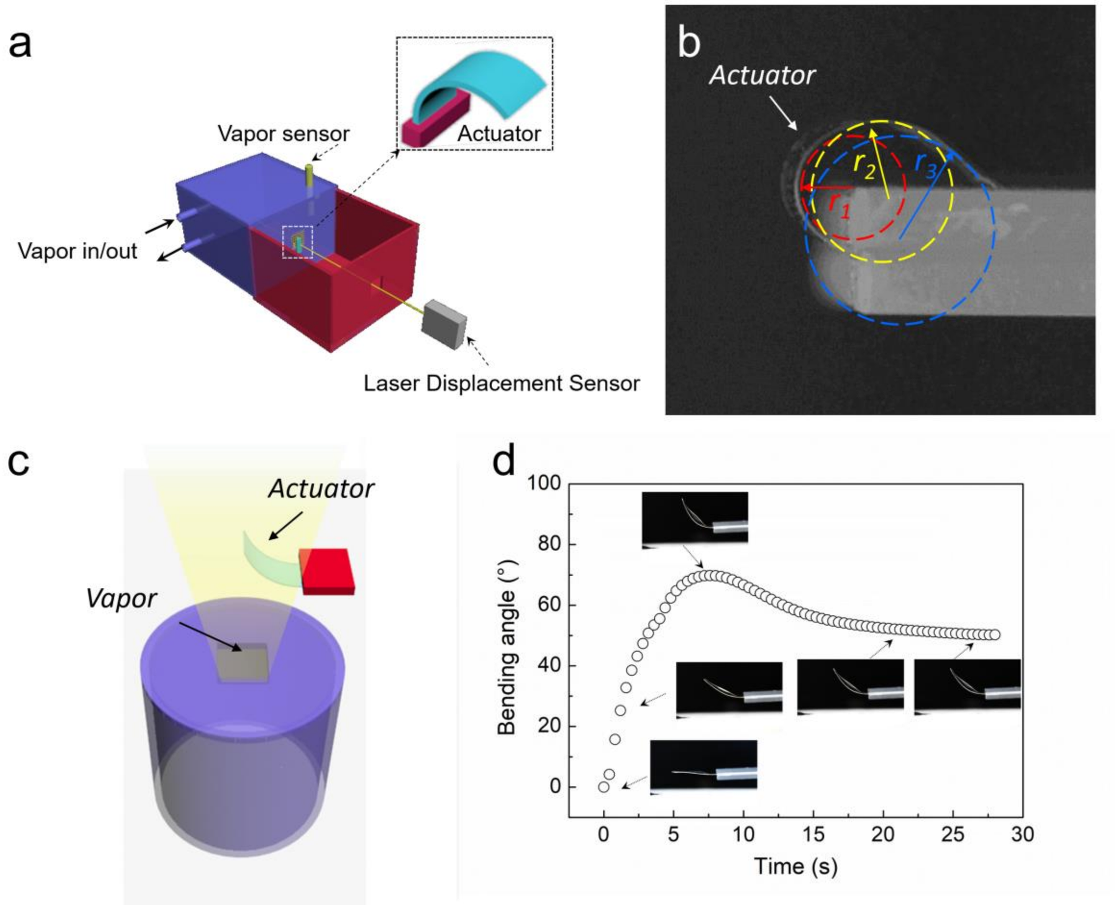

Supplementary Figure 6. Effect of ethanol vapor concentration on the curvature of monolayer PFSA membrane actuator. a Schematic illustration of the experimental measurement apparatus. **b** The scheme used for definition and calculation of curvature. (The three dashed circles (red, yellow and blue) represent the profile of the PFSA film. Here, *r* is the radius of every bent arch. The curvature was calculated by the equation below. *Curvature* = *1/r*). **c** Schematic illustration of the experimental measurement apparatus. **d** Single-layer PFSA actuator bending angle verses time when it was exposed in an ethanol vapor atmosphere which has concentration gradient.

Supplementary Table 2. Polarity and saturated vapor pressure of solvents.

Solvent	Polarity	Saturated vapor pressure (kPa)
Methanol	6.6	16.8
Ethanol	4.3	8.5
1-Butanol	3.7	0.82
1-propanol	4	5.8
water	4.2	3.16
Acetonitrile	6.2	12.15
Cyclohexane	0.1	13.3
Petroleum ether	0.01	53.3

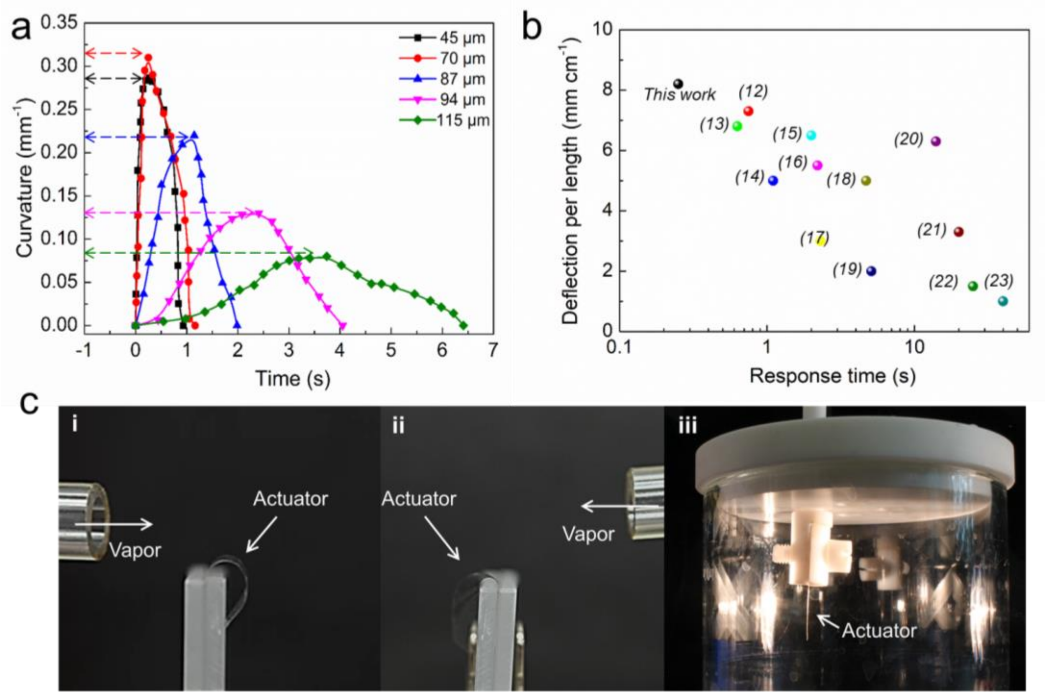

Supplementary Figure 7. Bending curvature change of single-layer PFSA films with different thickness. a Plots showing time dependence of the bending curvature change of single-layer PFSA films with different thickness upon exposure to 18% ethanol vapor conditions and relaxation dynamics after removing the ethanol vapor exposure. **b** Comparison of bending actuation properties and the response time in our study and previous bending actuators. [12-23] **c** The bending actuation behavior of single-layer PFSA triggered in different directions (i) trigger from left side, (ii) trigger from right side, (iii) trigger from two sides of the actuator.

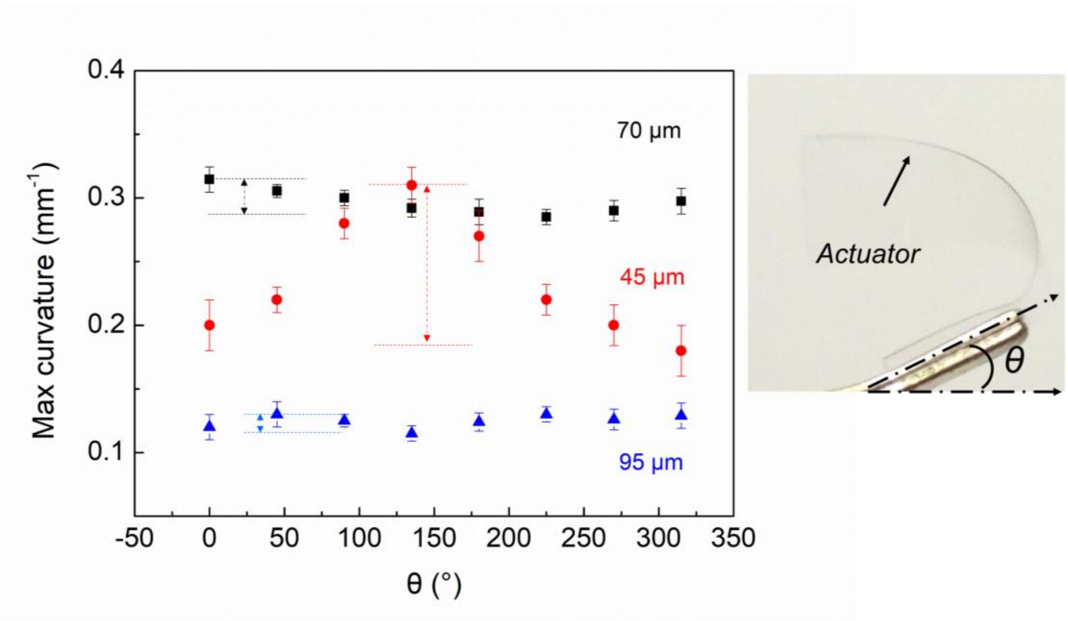

Supplementary Figure 8. Max curvature of monolayer PFSA films with different thicknesses versus angles to which the film was fixed relative to the horizontal plane. The right image presents a scheme providing the definition of θ.

Here, Films with three different film thicknesses, 45 μm, 70 μm, and 95 μm, were fixed at 8 different angles (0°, 45°, 90°, 135°, 180°, 225°, 270°, 315°) to the horizontal plane. We found that gravity certainly affects bending actuation performance, and the phenomenon is film thickness-dependent. For instance, the calculated value of bending curvature changed by more than 25% when 45 μm thick films were fixed at different angles. However, as the film thickness increased, the impact of gravity on the bending curvature decreased typically less than 5% percent when the thickness of the PFSA film actuator was 95 μm.

Supplementary Note1. The diffusion model and the relation between swelling and curvature

Consider the diffusion through the PFSA membrane with thickness *L*.

$$\frac{\partial c(x,t)}{\partial t} = D\frac{\partial^2 c(x,t)}{\partial x^2} \tag{1}$$

where *c* is the concentration function and *D* is the diffusion coefficient. Combined with the boundary conditions $c(x,t) = c_0$ at $x = 0$ and $c(x,t) = c_L$ at $x = L$, the transient solution of concentration profile for Supplementary Equation 1 is

$$c(x,t) = c_0 + \frac{c_L - c_0}{\mathrm{erf}\left(\frac{L}{\sqrt{4Dt}}\right)}\mathrm{erf}\left(\frac{x}{\sqrt{4Dt}}\right) \tag{2}$$

where erf (x) is the error function and is defined as $\mathrm{erf}\,(x) = \frac{2}{\pi}\int_0^x e^{-x^2}\,\mathrm{d}x$.

The concentration profiles $c(x)$ within the membrane of thickness *L* are curved until steady state has been reached, as shown in Supplementary Figure 9a. The curvature takes time to dissipate, in accord with Fick's Second Law (Supplementary Equation 1), until the pseudo-steady state is reached at about $t_4 = L^2/2D$ in Supplementary Figure 9a, where the concentration profile $c(x)$ is approximated as $c_0 - (c_0 - c_L)x/L$. The swelling strain distribution $\varepsilon(x)$ along the thickness of the membrane is assumed to be proportional to the concentration profile $c(x)$ as,

$$\varepsilon(x) = \alpha c(x,t) \tag{3}$$

where α is the swelling coefficient. In the experiments, exposure of one side of a PFSA membrane (thickness $L = 75$ μm) to ethanol vapor led to bending of the film and the maximum curvature was about 0.3 mm^{-1} (Supplementary Figure 9b), which can be seen in the pseudo-steady state, and the concentration profile $c(x)$ is approximated as $c^0 - (c^0 - c^L)x/L$. Thus the curvature κ in the pseudo-steady state can be expressed as,

$$\kappa = \alpha(c^0 - c^L)/L \tag{5}$$

To produce the bending actuation of the PFSA membrane with maximum curvature ~0.3 mm^{-1}, the needed swelling strain gradient $\alpha(c_0 - c_L)$ is about 2.3%, validated from both FEA simulation and theoretical analysis. With these experimental parameters $\alpha(c_0 - c_L) = 2.3\%$, and Supplementary Equation 2-3, a series of bending snapshots at $t = 0$ and t_1, t_2, t_3, and t_4 were simulated by FEA, where the bending curvature at $t = t_4$ is about 0.3 mm^{-1} and agrees with the experimental results.

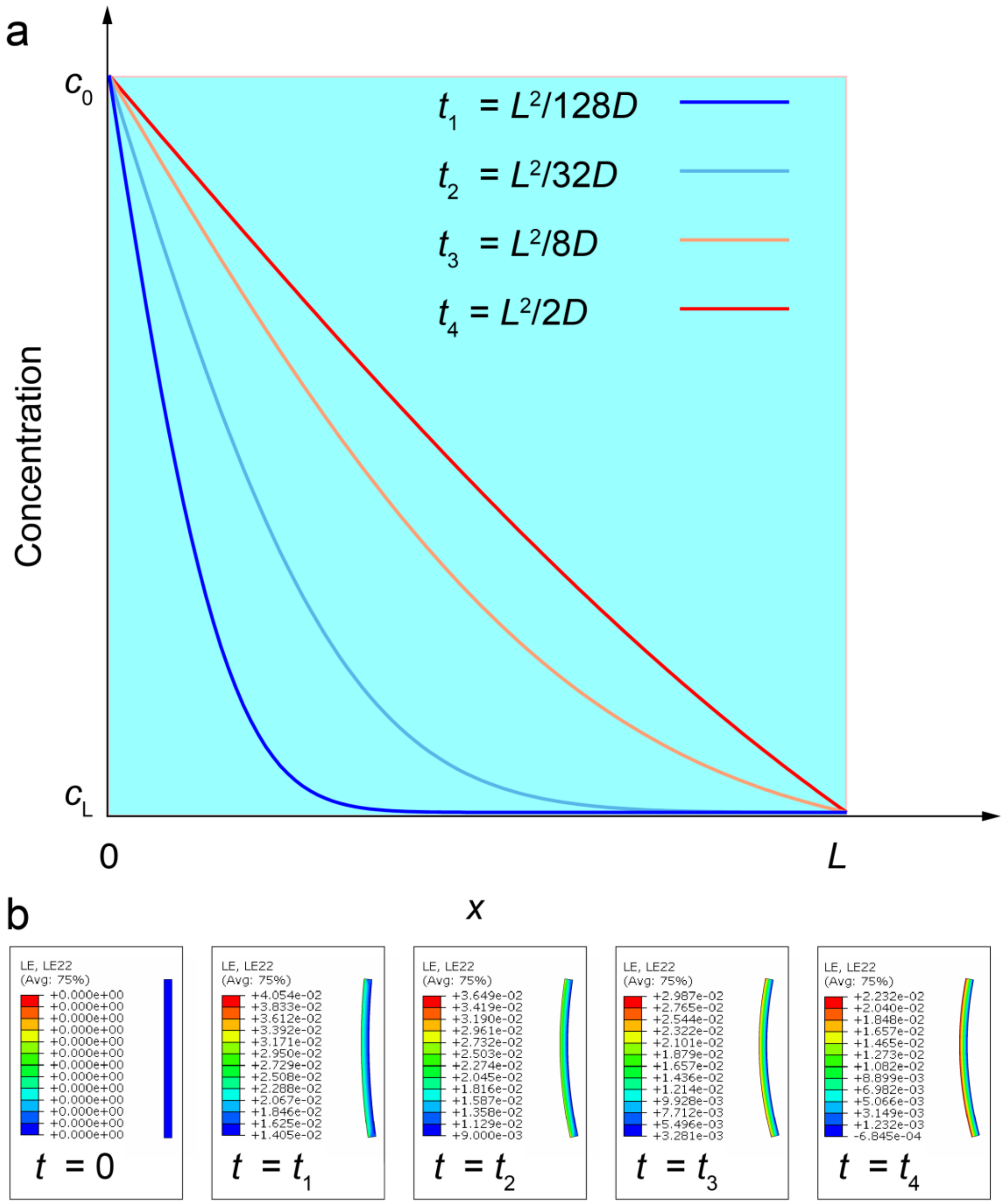

Supplementary Figure 9. FEA (Finite Element Analysis) results of strain distribution and calculated bending curvature. a Transient concentration profile in diffusion across the membrane with thickness L. The pseudo-steady state is approached at t of about $L^2/2D$, diagrammed as t_4, where the profile is an approximate straight line between $c(x,t) = c_0$ at $x = 0$ and $c(x,t) = c_L$ at $x = L$. **b** FEA simulation of a series of bending snapshots at $t = 0$ and t_1, t_2, t_3 and t_4, where the bending curvature at $t = t_4$ is about 0.3 mm^{-1}. It should be noted that the simulation is one segment of a PFSA membrane with a length of 1.5 mm and thickness 75 μm. (in experiments the length was 15 mm)

Supplementary Note 2. Comparison of the structure of films with different size of channels,

First, we assume the film has the same volume fraction of channels

$$\varphi = N\pi d^2 h/4V \tag{3}$$

where N is the number of channel with diameter d and length h, and V is volume of the film. The total interfacial functionalized area and channel length can be derived as

$$A = N\pi dh = 4V\varphi/d \tag{4}$$

and

$$L = Nh = 4V\varphi/\pi d^2, \tag{5}$$

Hence, the smaller d is, the larger A and L are. Furthermore, the larger functionalized area is in favor of adsorption of a gas molecule, and the higher surface-to-volume ratio is good for fast diffusion. Hence, these two factors play important roles in fast and large actuation associated with the molecular nano channel structure.

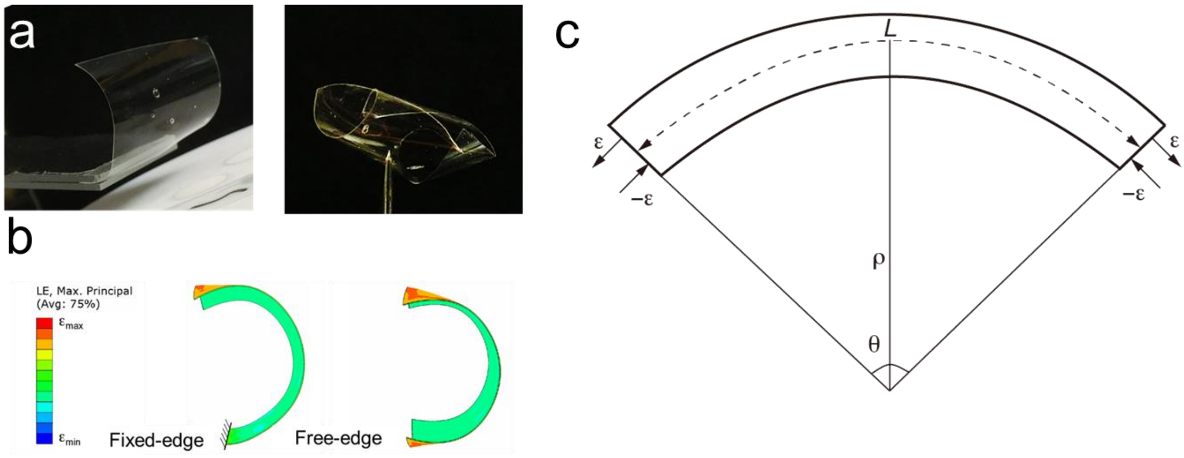

Supplementary Figure 10. The contribution of the effect of the clamped position and the structure of actuators (length-width ratio) to bending direction. The experiments **a** and simulations **b** shown the contribution of the effect of the clamped position and the structure of actuators (length-width ratio) to bending direction. **c** The actuation curvatures versus length-width ratio of monolayer one edge fixed PFSA film.

Here, for the single-layer PFSA actuator film, we used both the experiments and simulations to interrogate the contribution of the effect of the clamped position and the structure of actuators (length-width ratio) to bending direction. As shown in Supplementary Figure 10 a and b, the PFSA film unclamped actuator showed different bending behavior than that which was clamped because bending along the width direction is partially confined by the fixed edge. In addition to the effect of the clamp, the length-width ratio may also impact the degree of bending. In consideration of the length-width ratio, the bending deformation is more obvious along the long edge. Specifically, the radius of curvature ρ is the same if the swelling strain gradient is the same, and the bending angle $\beta = L/\rho$ is larger for long edge, where L is the length and β is the bend angle as shown in Supplementary Figure 10c. To clear highlight these two factors, in the manuscript, we have a larger length to width ratio for the sample clamped at one edge, so the bend along the width direction can be neglected.

The actuation property of the double-layer PFSA membrane actuator

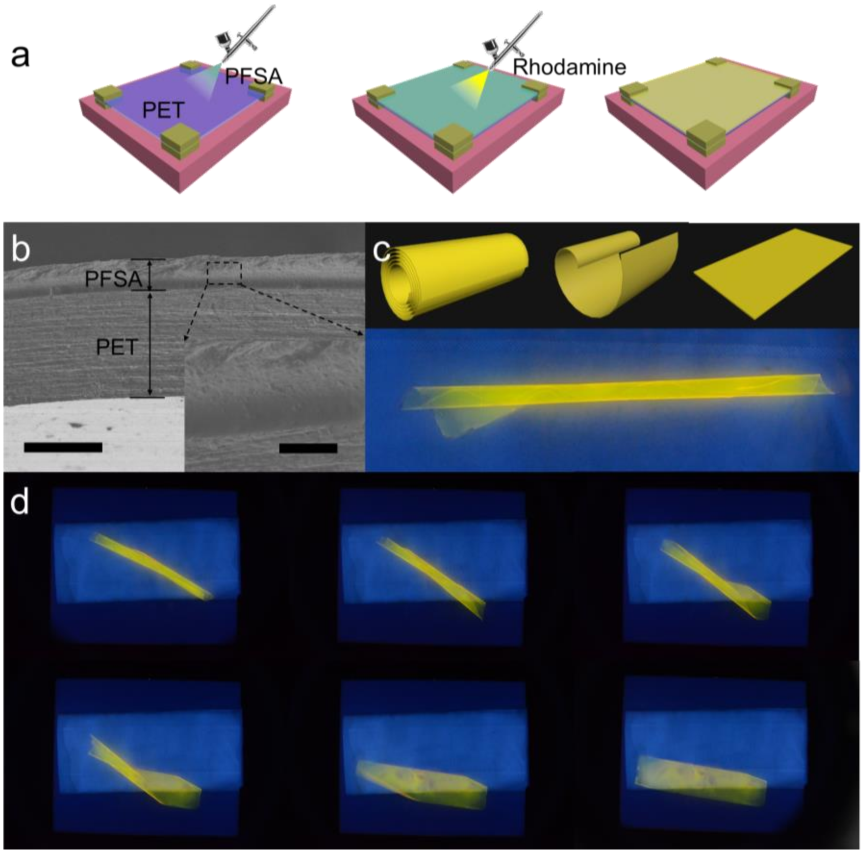

Supplementary Figure 11. The actuation performance of the bilayer PFSA actuator. a The scheme illustrating the preparation bilayer PFSA actuator. **b** SEM images showing cross-sectional images of the bilayer structure. **c, d** Schematic illustrations (top of c) and photographs (bottom of c and d) of spreading of process of the curly bilayer film, respectively.

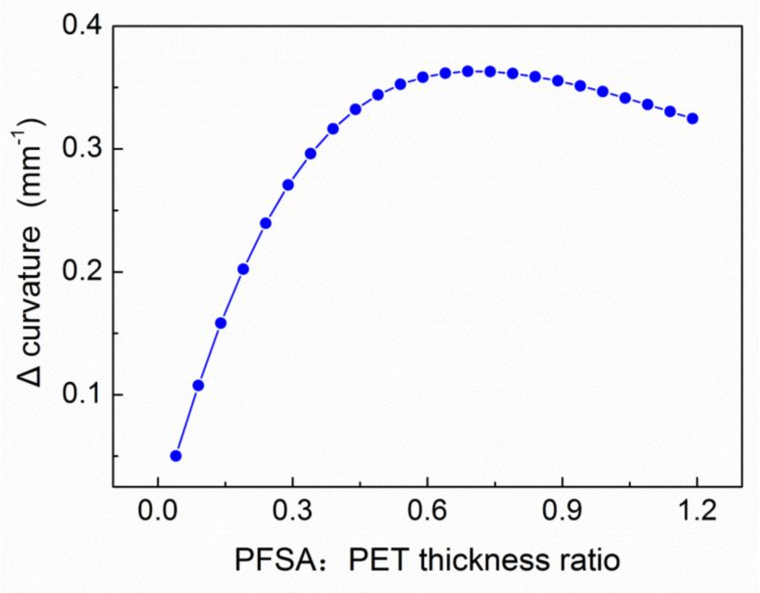

Supplementary Figure 12. The simulated curvature as a function of PFSA/PET thickness ratio.

The preparation of various patterns on the surface of the actuators

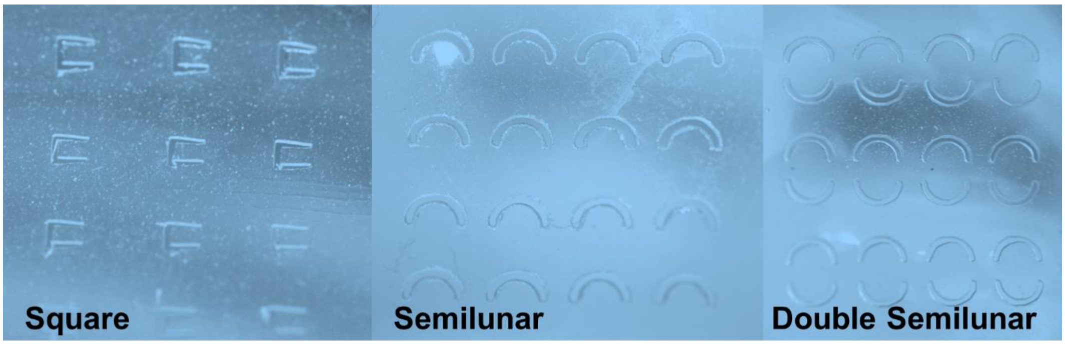

Supplementary Figure 13. Various patterns on the surface of the actuators.

Interactive mechanochromic actuator through CQA surface modification

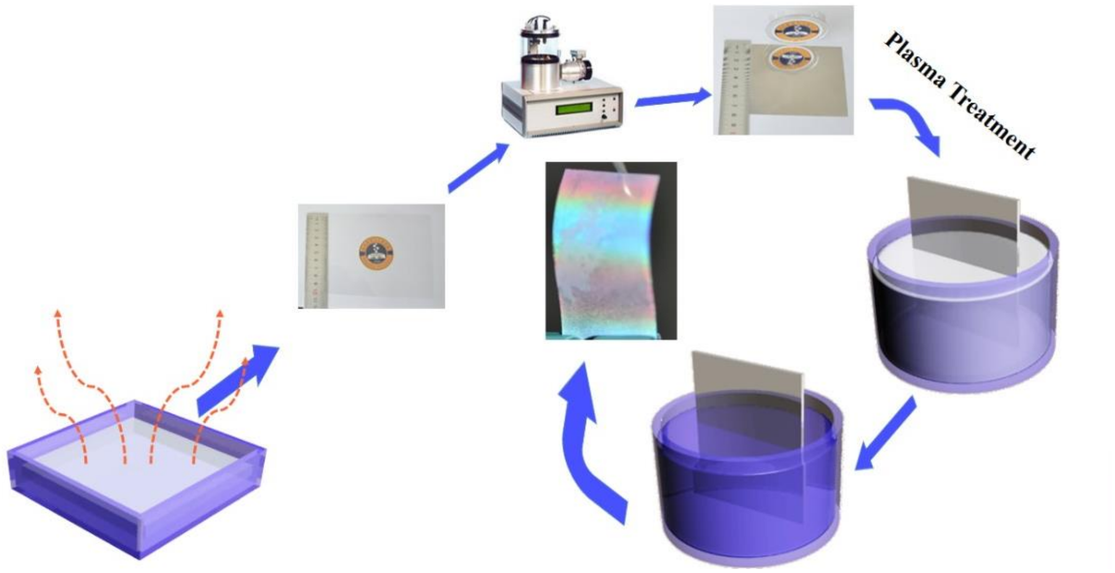

Supplementary Figure 14. Over view of the fabrication process of stimuli-responsive color change actuator.

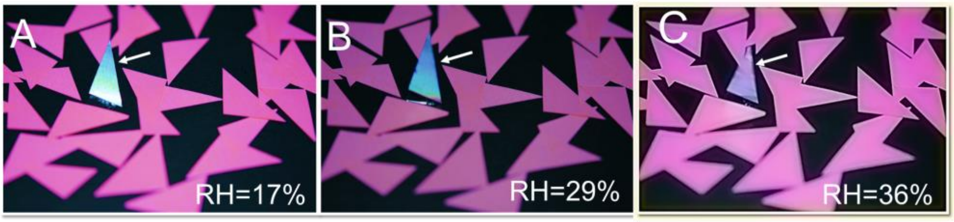

Supplementary Figure 15. A demonstration of the stimuli-responsive color change actuator as invisible shield material. Images show color changes induced by the change of relative humidity (RH).

Supplementary Reference：

1 Zhao, Q. *et al.* An instant multi-responsive porous polymer actuator driven by solvent molecule sorption. *Nat. Commun.* **5,** 4293 (2014).

2 Liu, Y. *et al.* Humidity-and Photo-Induced Mechanical Actuation of Cross-Linked Liquid Crystal Polymers. *Adv. Mater.* **29**, 1604792 (2017).

3 Zhang, L., Chizhik, S., Wen, Y. & Naumov, P. Directed motility of hygroresponsive biomimetic actuators. *Adv. Funct. Mater.* **26**, 1040-1053 (2016).

4 Zhang, L., Naumov, P. e., Du, X., Hu, Z. & Wang, J. Vapomechanically Responsive Motion of Microchannel-Programmed Actuators. *Adv. Mater.* **29**, 702231 (2017).

5 Lin, H. *et al.* Flexible and Actuating Nanoporous Poly (Ionic Liquid)–Paper-Based Hybrid Membranes. *ACS. Appl. Mater. Inter.* **9**, 15148-15155 (2017).

6 Shen, L., Fu, J., Fu, K., Picart, C. & Ji, J. Humidity responsive asymmetric free-standing multilayered film. *Langmuir* **26**, 16634-16637 (2010).

7 Dai, M. *et al.* Humidity-responsive bilayer actuators based on a liquid-crystalline polymer network. *ACS. Appl. Mater. Inter.* **5**, 4945-4950 (2013).

8 He, S. *et al.* A Mechanically Actuating Carbon-Nanotube Fiber in Response to Water and Moisture. *Angew. Chem. Int. Edit.* **127**, 15093-15097 (2015).

9 Di, J. *et al.* Strong, Twist-Stable Carbon Nanotube Yarns and Muscles by Tension Annealing at Extreme Temperatures. *Adv. Mater.* **28**, 6598-6605 (2016).

10 Kim, S. H. *et al.* Bio-inspired, moisture-powered hybrid carbon nanotube yarn muscles. *Sci. Rep.* **6**, 23016 (2016).

11 Jiang, Y. *et al.* Spontaneous, Straightforward Fabrication of Partially Reduced Graphene Oxide–Polypyrrole Composite Films for Versatile Actuators. *ACS Nano* **10**, 4735-4741 (2016).

12 Deng, J. *et al.* Tunable photothermal actuators based on a pre-programmed aligned nanostructure. *J. Am. Chem. Soc.* **138**, 225-230 (2016).

13 Zhang, X. *et al.* Photoactuators and motors based on carbon nanotubes with selective chirality distributions. *Nat. Commun.* **5,** 2983 (2014).

14 Wu, W. *et al.* NIR-light-induced deformation of cross-linked liquid-crystal polymers using upconversion nanophosphors. *J. Am. Chem. Soc.* **133**, 15810-15813 (2011).

15 Zhang, X. *et al.* Optically-and thermally-responsive programmable materials based on carbon nanotube-hydrogel polymer composites. *Nano. Lett.* **11**, 3239-3244 (2011).

16 Yoshino, T. *et al.* Three-dimensional photomobility of crosslinked azobenzene liquid-crystalline polymer fibers. *Adv. Mater.* **22**, 1361-1363 (2010).

17 Lan, T., Hu, Y., Wu, G., Tao, X. & Chen, W. Wavelength-selective and rebound-able bimorph photoactuator driven by a dynamic mass transport process. *J. Mater. Chem. C* **3**, 1888-1892 (2015).

18 Wang, E., Desai, M. S. & Lee, S.-W. Light-controlled graphene-elastin composite hydrogel actuators. *Nano. Lett.* **13**, 2826-2830 (2013).
19 Jiang, W. *et al.* Photoresponsive Soft-Robotic Platform: Biomimetic Fabrication and Remote Actuation. *Adv. Funct.l Mater.* **24**, 7598-7604 (2014).
20 Li, Q. *et al.* Large-strain, multiform movements from designable electrothermal actuators based on large highly anisotropic carbon nanotube sheets. *ACS Nano* **9**, 409-418 (2015).
21 Sun, X. *et al.* Unusual reversible photomechanical actuation in polymer/nanotube composites. *Angew. Chem. Int. Edit.* **51**, 8520-8524 (2012).
22 Sahoo, S. C. *et al.* Actuation based on thermo/photosalient effect: a biogenic smart hybrid driven by light and heat. *RSC. Adv.* **4**, 7640-7647 (2014).
23 Lu, S. & Panchapakesan, B. Optically driven nanotube actuators. *Nanotechnology* **16**, 2548 (2005).
