# Supplementary material for: Molecular-channel driven actuator with considerations for multiple configurations and color switching
Source: Nat Commun. 2018 Feb 9;9:590. doi: 10.1038/s41467-018-03032-2 (PMC5807312; doi:10.1038/s41467-018-03032-2)
Supplement: Supplementary file 3 — Description of Additional Supplementary Files [file 41467_2018_3032_MOESM3_ESM.pdf]

Description of Additional Supplementary Files

File Name: Supplementary Movie 1
Description: In-situ time-dependent 2D GIWAXS patterns using 10 seconds interval.

File Name: Supplementary Movie 2
Description: The adaptive actuation movement of a PFSA membrane (5 mm×15 mm×75 μm) in response to ethanol vapor sorption and desorption.

File Name: Supplementary Movie 3
Description: The time-dependent untwisting actuation of the helical strip before and after triggering by moisture.

File Name: Supplementary Movie 4
Description: The self-adaptive actuation movement of the actuator array before and after triggering by moisture.

File Name: Supplementary Movie 5
Description: FEA results of the actuator array self-adaptive actuation.
